# Lower Broadly Neutralizing Antibody Responses in Female Versus Male HIV-1 Infected Injecting Drug Users

**DOI:** 10.3390/v11040384

**Published:** 2019-04-25

**Authors:** Zelda EULER, Tom L. VAN DEN KERKHOF, Roger D. KOUYOS, Damien C. TULLY, Todd M. ALLEN, Alexandra TRKOLA, Rogier W. SANDERS, Hanneke SCHUITEMAKER, Marit J. VAN GILS

**Affiliations:** 1 Department of Experimental Immunology, Amsterdam UMC, University of Amsterdam, 1105 AZ Amsterdam, The Netherlands; zeuler@its.jnj.com (Z.E.); tom.vdkerkhof@gmail.com (T.L.V.D.K.); HSchuite@its.jnj.com (H.S.); 2 Department of Medical Microbiology, AMC, Amsterdam UMC, University of Amsterdam, 1105 AZ Amsterdam, The Netherlands; r.w.sanders@amc.uva.nl; 3 Institute of Medical Virology, University of Zurich, CH-8057 Zurich, Switzerland; roger.kouyos@uzh.ch (R.D.K.); trkola.alexandra@virology.uzh.ch (A.T.); 4 Division of Infectious Diseases and Hospital Epidemiology, University Hospital Zurich, CH-8091 Zurich, Switzerland; 5 Ragon Institute of MGH, MIT and Harvard, Cambridge, MA 02139, USA; tully.damien@mgh.harvard.edu (D.C.T.); tallen2@partners.org (T.M.A.); 6 Department of Microbiology and Immunology, Weill Medical College of Cornell University, New York, NY 10065, USA

**Keywords:** HIV-1, transmission route, injecting drug users, broadly neutralizing antibodies, gender

## Abstract

Understanding the factors involved in the development of broadly neutralizing antibody (bNAb) responses in natural infection can guide vaccine design aimed at eliciting protective bNAb responses. Most of the studies to identify and study the development of bNAb responses have been performed in individuals who had become infected via homo- or heterosexual HIV-1 transmission; however, the prevalence and characteristics of bNAb responses in injecting drug users (IDUs) have been underrepresented. We retrospectively studied the prevalence of bNAb responses in HIV-1 infected individuals in the Amsterdam Cohort, including 50 male and 35 female participants who reported injecting drug use as the only risk factor. Our study revealed a significantly lower prevalence of bNAb responses in females compared to males. Gender, transmission route and CD4+ count at set point, but not viral load, were independently associated with the development of bNAb responses in IDUs. To further explore the influences of gender in the setting of IDU, we also looked into the Swiss 4.5k Screen. There we observed lower bNAb responses in female IDUs as well. These results reveal that the emergence of bNAbs may be dependent on multiple factors, including gender. Therefore, the effect of gender on the development of bNAb responses is a factor that should be taken into account when designing vaccine efficacy trials.

## 1. Introduction

An effective HIV-1 vaccine should be capable of eliciting broadly neutralizing antibodies (bNAbs), defined as the ability to neutralize various heterologous viruses from different subtypes, in order to provide protection against HIV-1 acquisition [1,2,3]. During HIV-1 infection, neutralizing antibodies (NAbs) develop within the first three months of infection [4,5]. However, these NAbs are usually strain-specific and the autologous virus can rapidly escape from them. bNAbs develop within 1–3 years post-seroconversion (post-SC), but only in about 10–30% of HIV-1 infected individuals [6,7,8,9,10,11,12,13,14,15,16,17]. Approximately 1% of the HIV-1 infected individuals, termed “elite neutralizers”, develop bNAbs that neutralize the majority of HIV-1 subtypes with very high breadth and potency [8,9,12,14,15]. Although bNAbs do not protect from disease progression, the passive transfer of bNAbs can completely block infection by a chimeric simian–human immunodeficiency virus (SHIV) in nonhuman primate studies [18,19,20,21,22,23,24] and reduce viral load in chronically infected humans and macaques [25,26,27,28,29]. The presence of bNAbs in humans indicates that there are no fundamental immunological barriers to prevent their induction, lending further support to the search for a vaccine that induces bNAbs.

The most predictable clinical markers for the development of bNAbs are duration of infection, high viral load, and in some cohorts low CD4+ T cell count [6,7,11,14,15,16,17,30,31]. Furthermore, circulatory follicular helper CD4+ T cells (Tfh cells) [32], as well as HIV-specific Tfh cells in the lymph and some human leukocyte antigen (HLA) class II alleles are associated with bNAb development [33,34]. Virological markers such as viral diversity, HIV-1 subtype, antibody effector functions, IgG-subclass and particular envelope glycoprotein (Env) characteristics are also suggested to be potential contributors to the development of neutralization breadth [12,15,35,36,37,38]. On the other hand, history of antiretroviral use, age, and transmission route did not correlate with the development of bNAbs in previous studies [11,15,39]. Interestingly, in the Swiss 4.5K Screen Rusert et al. [15] found a positive correlation for duration of infection and black ethnicity with the development of bNAbs.

Most of the studies to identify bNAb responses were performed in individuals who were infected via homo- (men who have sex with men, MSM) or heterosexual HIV-1 transmission. The determinants of bNAb induction in injecting drug users (IDUs) remains underrepresented, while the immunomodulatory effect of drug use [40,41,42,43] and the higher risk of multiple virus transmissions may influence the development of bNAbs [38]. Here, we studied the prevalence and potency of bNAb responses in a mixed-gender cohort of HIV-1 infected individuals who reported injecting drug use as their only HIV-1 risk factor. The data were compared to similar data obtained from MSM participants of the Amsterdam Cohort [6,31,39], as well as MSM and IDU of the Swiss 4.5K Screen [15].

## 2. Materials and Methods

### 2.1. Ethics Statement

The Amsterdam Cohort Studies on HIV-1 infection and AIDS (Amsterdam Cohort) are being conducted in accordance with the ethical principles set out in the declaration of Helsinki, and all participants provided written informed consent. The study was approved by the institutional Medical Ethics Committee of the Academic Medical Center, University of Amsterdam.

Data from the Swiss 4.5 Screen integrated as a comparison group in the current study comprised solely the re-analysis of previously generated data [15,38]. Ethical approval from the Swiss HIV Cohort Study (SHCS) and the Zurich Primary HIV Infection Study and written informed consent from all participants has been obtained as detailed in [15].

### 2.2. Study Population and Phenotype

We screened serum samples from participants of the Amsterdam Cohort for the presence of bNAb responses. The study population consisted of a total of 299 HIV-1 infected MSM [39,44] and 85 HIV-1 infected IDUs (50 men and 35 women) [45,46]. Participants were eligible to participate in this study when they were therapy-naïve and when a serum sample was available ~3 years post imputed or documented date of seroconversion (SC), when bNAb activity commonly peaks [6,7,8,10,11,14,17]. For MSM, this was on average 34 months (range, 21–37 months) and for IDUs, on average 36 months (range, 23–55 months). A total of 1380 MSM and 672 IDU, of which 243 were female, were selected from the Swiss 4.5 Screen, established as previously described [15,38].

### 2.3. Neutralization Assays

Sera were tested for bNAb responses in a pseudovirus assay involving six tier-2 viruses (JRCSF, 92BR020, 93IN905, 92TH021, 94UG103 and MGRMC026) in a single round of viral infection developed by Monogram Biosciences. This six-virus panel covered 93% of the variation in neutralization of a larger pseudovirus panel (*n* = 15) [8]. For each individual, we calculated the geometric mean ID50 titer (GMT) across the six-virus panel. Data on HIV-1 neutralizing activity in sera of MSM were available from our previous studies [6,31,39], and control sera were measured in each assay for comparability. Neutralization breadth in the Swiss 4.5k Screen was measured against an eight-virus multi-clade panel in a pseudovirus assay on TZM-bl cells as previously described [15,38].

### 2.4. Statistical Analysis

Differences between groups were analyzed with a Mann–Whitney test. Mann–Whitney tests and Spearman correlation tests were performed in GraphPad prism 7 (GraphPad Software, La Jolla, California, USA). A univariate and multivariate regression analysis on both the IDU and MSM cohort was performed using SPSS with the logarithmic transformed GMT as dependent factor and mode of transmission, gender, viral load at setpoint, and CD4+ T cell count at setpoint as potential predictors. Mode of transmission and gender were grouped as MSM male, IDU male and IDU female. As the MSM cohort did not include females, gender and mode of transport could not be separated as independent variables. The effect of bNAb responses on disease progression was analyzed in a Kaplan–Meier and Cox proportional hazard analysis using clinical AIDS (1993 CDC definition) as an endpoint. Individuals were divided into 3 groups: those who neutralized ≤ 1; 2 or 3; or ≥ 4 viruses at an ID50 titer ≥ 100. Left truncation of follow-up time was performed for the time between the imputed SC date and first seropositive visit using S-Plus 8 (Insightful Corporation, Seattle, Washington, USA). P-values < 0.05 were considered significant.

In order to determine the effect of IDU-transmission route on bNAb activity amongst participants in the Swiss 4.5 k Screen, we used uni- and multivariable Tobit regression models with the neutralization score determined previously [15] as an outcome variable. These models are appropriate for the neutralization score data since they consider the truncated nature of these scores. In analogy to the studies using the Amsterdam Cohort, we focused in this analysis on MSM and IDUs (i.e., other transmission groups were excluded). Since the vast majority of MSM and IDUs in the SHCS from which Swiss 4.5K Screen participants were selected are of white ethnicity (93.3%) and infected with subtype B (92.5%), we restricted this analysis to individuals of white ethnicity infected with subtype B. The multivariable model was adjusted for duration of infection, viral load, and CD4+ T cell count as these variables were found to be associated with neutralization breadth by Rusert et al. [15].

### 2.5. Diversity Analysis

The HIV-1 envelope gp160 gene (*env*) was PCR amplified from DNA isolated from PBMCs that were infected in vitro with a single clonal HIV-variant and subsequently sequenced as described previously [47]. Nucleotide sequences were aligned using ClustalW in the software package of BioEdit. Nucleotide diversity within each individual was calculated for 23 MSM and 15 IDU infected individuals with median GMTs of 64 (range 20–782) and 47 (range 23–978), respectively, with the Kimura-2 parameter substitution model in the software package MEGA 6. The selection of 23 MSM and 15 IDU infected individuals was made on the basis that *env* sequences from within the first year post-SC from these individuals were available. To assess if multiple virus transmission (MVT) has occurred in the IDU and MSM, we analyzed the earliest available *env* sequences, within 3 months of SC (MSM = 8 and IDU = 5), as previously described [48,49,50,51].

## 3. Results

### 3.1. Broadly Neutralizing Antibody Responses in Injecting Drug Users

**Figure 1:**
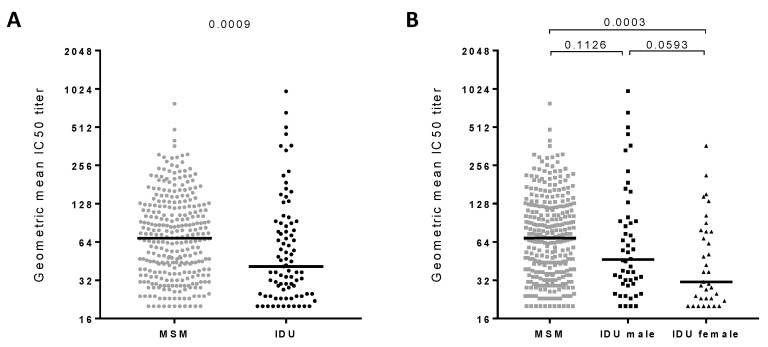
The potency of broadly neutralizing antibodies (bNAb) in the HIV-1 infected men who have sex with men (MSM) and injecting drug user (IDU) participants of the Amsterdam Cohort. Serum samples from MSM (*n* = 299) and IDUs (*n* = 85) from the Amsterdam Cohort Studies on HIV-1 infection and AIDS (ACS) were screened for the capability to neutralize six viruses from different HIV-1. (**A**) Comparison of bNAb responses in MSM (gray data points) and IDUs (black data points). Each data point represents one individual’s geometric mean ID50 titer (GMT) across the six-virus panel. (**B**) Same as A, but with the IDU separated in men (square) and women (triangle). Each data point represents one individual’s GMT. Statistically significant differences between the different cohorts were determined using a Mann–Whitney t-test and the respective P-values are shown. Horizontal bars represent the median values per group.

The prevalence and potency of bNAb responses was determined in the IDU participants of the Amsterdam Cohort (*n* = 85) and compared with the MSM participants of the Amsterdam Cohort (*n* = 299; previously determined [6,31,39]) (Figure 1 and Supplementary Table S1) analyzed against the same virus panel. The potency of the bNAb responses of all participants was defined by the geometric mean titer (GMT) values across the six-virus panel and was strongly correlated with both the number of viruses neutralized (Spearman r = 0.85, P < 0.001) as well as the number of viruses that were neutralized with neutralization titers > 100 (Spearman r = 0.92, *p* < 0.001). Of the 384 HIV-1 infected individuals (IDU and MSM combined), 25% developed bNAb responses, defined by their ability to neutralize ≥ 4 viruses of the six-virus panel, at ID50 titers > 100. The prevalence of bNAbs in the IDUs was lower compared to the prevalence in the MSM (19% and 27%, respectively; Table 1). Furthermore, the bNAb responses in IDUs were weaker as they had significantly lower GMT values compared to MSM (*p* = 0.0009) (Figure 1A). The IDUs showed an abnormal distribution of GMT values, with a number of outliers at the top of the range (median GMT = 41 (range 20–978)), whereas the MSM had a more normal distribution of the GMT values (median GMT = 68 (range 20–782)). Interestingly, the IDUs had significantly more elite neutralizers (GMT > 500) compared to the MSM (3.5% and 0.3%, respectively; *p* = 0.035, Fisher’s exact test) (Table 1).

As the IDU participants are a mixed-gender population, we repeated our analyses after the exclusion of women (*n* = 35), which allowed a comparison of exclusively men of both the IDUs (*n* = 50) and MSM (*n* = 299) (Figure 1B). After the exclusion of women, the difference in GMT and prevalence of bNAb responses between the two cohorts faded (*p* = 0.1126). This suggested that the female gender contributed to the differences between the IDU and MSM in the Amsterdam Cohort. Therefore, we compared the GMT values between all the men (*n* = 349) and women (*n* = 35), irrespective of the route of HIV-1 transmission. HIV-1 infected men had higher GMT values than HIV-1 infected women (*p* = 0.0005). A similar trend was observed within the IDU cohort (Figure 1B) (*p* = 0.0593); however, this was not statistically significant, most likely due to the low number of participants per group. Furthermore, male IDUs were more frequently classified as elite neutralizers than female IDUs (3/50 or 6% versus 0/35 or 0%, respectively; Table 1).

### 3.2. Clinical Factors Associated with the Development of Broadly Neutralizing Antibody Responses

**Figure 2:**
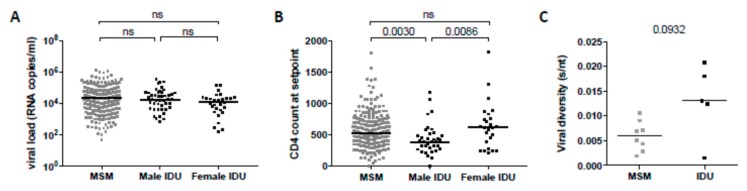
Clinical factors associated with the development of broadly neutralizing antibody responses in men and women in the MSM and IDU Amsterdam Cohort. Comparison between MSM, male IDUs and female IDUs for (**A**) viral load in plasma at setpoint and (**B**) CD4+ T cell counts at setpoint. Each data point represents one individual. Statistically significant differences between the different groups were determined using a Mann–Whitney t-test and the P-values are shown; ns, not significant. Horizontal bars represent the median value per group. (**C**) Early sequence *env* diversity is shown in substitutions/nucleotides (s/nt) for five IDU male versus eight MSM infected individuals. *env* sequences were obtained within the first three months post-SC. Each dot represents one individual and horizontal bars represent the median diversity per group.

Duration of infection was shown to be a very important predictor for bNAb development. In our study, we included patients and samples based on similar time points post-SC (~3 years post-SC), and therefore we did not observe a correlation between duration of infection and GMT. In addition to duration of infection, high viral load and low CD4+ T cell count were the strongest predictors for the development of bNAb responses in previous studies [6,7,11,14,15,16,17,31,33]. We analyzed the association between the CD4+ T cell count and the viral load at setpoint and the development of bNAbs within the MSM and the IDU participants. We observed that within the IDUs, women had higher mean CD4+ T cell counts, although not significantly higher than the MSM, while the viral load at setpoint was not statistically different between men and women in the IDUs (Figure 2A,B). In line with this, in the combined MSM and IDU, we observed a negative correlation between the CD4+ T cell count at setpoint (approximately 18 months post-SC) (Spearman r = −0.20, *p* < 0.001), and a positive correlation between the viral load at setpoint, and the development of bNAbs (Spearman r = 0.11, *p* = 0.031). These correlations were stronger when only the MSM were analyzed (Spearman r = −0.24, *p* < 0.001 for CD4+ T cell count; Spearman r = 0.14, *p* = 0.014 for viral load). Conversely, in the IDUs, a similar trend was observed between the CD4+ T cell count at setpoint and bNAb responses (Spearman r = −0.20, *p* = 0.133), while no correlation with viral load at setpoint was observed, which could be due to the low number of participants. To determine whether these parameters were independently associated with the development of bNAb responses, we performed a multivariate model analysis on the combined IDU and MSM cohorts using all parameters as covariates (Table 2). As the MSM consisted of males only, precluding the evaluation of the mode of transmission as an independent variable, we combined the variables of gender and transmission into the following groups: MSM, male IDU and female IDU. In the multivariate model, the viral load at setpoint was no longer independently associated with bNAb development. However, low CD4+ T cell count at setpoint and male gender combined with mode of transmission were still associated with the development of bNAbs (*p* = 0.017 and *p* = 0.001, respectively).

bNAb responses generally have no effect on disease progression [6,52]. Consistent with these reports, we found that individuals in the IDU cohort that could neutralize the majority of the viruses (≥4) at ID50 titers higher than 100 (*n* = 16) had a similar time to AIDS compared to IDU with intermediate NAb breadth (neutralizing 2 or 3 viruses, *n* = 24) or those with no bNAb responses (neutralizing 1 or none of the six viruses, *n* = 45). The results indicate that bNAbs in IDUs also had no beneficial effect on the clinical course of infection, similar to bNAbs in individuals who were infected via (MSM) or heterosexual transmission [6,16,31,36].

Viral diversity has been shown to correlate with the development of bNAb responses [15,36]. Therefore, we performed a variety of approaches to assess whether early sequence diversity, including multiple virus transmission (MVT), was linked to bNAb development. We did not observe any correlation between the presence of bNAbs and the *env* diversity in these individuals. However, we found that the sequence diversity of viruses isolated within the first three months post-SC may be higher in the IDUs compared to the MSM. Although the numbers of individuals eligible for inclusion in these analyses was small and the trend did not reach statistical significance (P = 0.0932), it may point at possible MVT in (some of) the IDUs (Figure 2C). Interestingly, four out of five IDU and only one out of eight MSM displayed high *env* diversity (>0.01) and demonstrated phylogenetic evidence of MVT (*p* = 0.022). Nevertheless, we could not find significant evidence for an association between viral diversity and bNAb development. However, we note that the number of individuals for which early *env* sequences were available and the number of sequences per timepoint were too small to study this reliably.

### 3.3. Broadly Neutralizing Antibody Responses in the Swiss 4.5K Screen

Previous studies on clinical variables associated with the development of bNAb responses did not show a strong association with the gender of the infected individual [8,11,14,15,53,54,55,56]. A lower frequency of neutralization breadth was, however, detected amongst females in the Swiss 4.5k Screen [15,38]. To further explore the influences of gender in the setting of IDU, we therefore re-analyzed the neutralization data of 1380 MSM and 672 IDU, of which 243 were female, established by Rusert et al. [15]. The direct comparison of MSM, male and female IDU (Table 3), analogous to the analysis of the Amsterdam Cohort (Table 2), showed that also in the Swiss 4.5K Screen, female IDUs had significantly lower neutralization scores than male IDUs. Notably, in contrast to the Amsterdam Cohort, IDUs showed higher breadth compared to MSM. This highlights that IDU status is linked with positive and female gender with negative drivers of neutralization breadth in the Swiss 4.5K Screen. While we observed a significant negative association between CD4+ T cell count and neutralization score in the univariable model (Table 3), the effect was lost in the multivariable model, likely reflecting the inverse relationship between CD4 cell numbers and viral load in the Swiss 4.5K Screen. Finally, we observed that neutralization scores were strongly and significantly associated with both infection time and virus load (Table 2), which is in line with previous studies [8,11,14,15,53,54,55,56]. Time of infection was not a factor in the Amsterdam Cohort dataset, because samples were included on very similar time post-SC (~3 years post-SC). Overall, these data confirm the lower neutralization breadth in female IDU compared to male IDU observed in the Amsterdam Cohort.

## 4. Discussion

We found that individuals in the Amsterdam Cohort infected with HIV-1 via contaminated needles generally had lower bNAb titers compared to HIV-1 infected MSM. This finding was not related to the calendar period in which the participants were infected or the subtype with which they were infected, because the MSM and IDU participants of the Amsterdam Cohort are similar in both respects (infection between 1982 and 1997 and exclusive infection with subtype B for both MSM and IDUs). Moreover, no significant differences in age, disease course, ethnicity and viral loads were observed between the IDU and MSM participants, which could explain the difference in bNAb development between these two groups. Interestingly, this difference appears to be independently associated with female gender in the IDU group, with females having lower bNAb responses compared to male IDUs. Previous studies have not observed such a difference when male versus female heterosexual HIV-1 transmission was studied [6,11,16,17,31]. These studies were conducted on different subgroups and at different infection times; however, in one study, the frequency of antibody breadth was lower in females [15].

The most predictable clinical markers for the development of bNAbs observed in previous studies are a high viral load and a reduced CD4+ T cell count [6,11,16,17,31]. Interestingly, one study showed that both IDUs and women in general have a higher CD4+ T cell count at SC compared to MSM [57], which have been shown to be predictors of lower bNAb responses. In our current study, the overall CD4+ T cell count and viral load at setpoint of the Amsterdam MSM and IDU participants were similar and confirmed to be correlated with the development of bNAb responses. Moreover, CD4+ T cell count at setpoint and combined gender and transmission route, but not viral load at setpoint, predicted the presence of bNAb in the multivariate analysis. Interestingly, within the Amsterdam IDU participants, we observed higher CD4+ T cell counts for women compared to men, which could be correlated to the lower bNAb responses observed in female IDUs. However, the CD4+ T cell counts for the female IDUs were similar to the MSM CD4+ T cell count at setpoint, contradicting the idea that CD4+ T cell count is the most important marker for bNAb development. Also, because CD4+ T cell count at setpoint, combined gender and transmission route and viral load at setpoint were all independently associated with bNAbs, the development of bNAbs remains multifactorial.

In the Swiss 4.5K Screen, female IDUs had significantly lower neutralization scores than male IDUs, who displayed higher neutralization activity than MSM, suggesting a positive influence of the IDU status in men but not women on bNAb development. A higher neutralization activity amongst male IDUs was not observed in the Amsterdam Cohort. The differences in bNAb responses between MSM and male IDU within the two cohorts, strengthen the observation that female gender was the strongest association for lower bNAb responses. Notably, in contrast to the Amsterdam Cohort, no strong effect of CD4+ T cell counts on bNAb activity were seen in the Swiss 4.5K Screen, as the negative association of CD4 cell counts with bNAb responses was lost in multivariable testing. Some of these individual differences may be in part due to differential cohort size and design. For instance, as the Swiss 4.5K screen recruited individuals at different stages of the infection, whereas the Amsterdam IDU cohort was selected from a narrower range, the predictive capacity of CD4 and viral load may differ. Despite the individual differences, the two cohorts strengthen the key observation that female IDUs have lower bNAb responses compared to male IDUs, indicating that there is a fundamental difference in bNAb development between male and female IDU. Interestingly, in most previous studies, this gender bias was not observed in women with heterosexual transmission [8,11,14,53,54,55,56].

Recreational drugs can have various immune-modulating properties depending on the frequency of use and type of drug used [41,42]. For example, it has been suggested that endogenous opioids can suppress B cell proliferation [43]. How this might influence bNAb development and whether drugs might have differential effects in males and females is unknown. In addition, we could not exclude any cofounder effects of alcohol or other polysubstance use, as this was not systematically recorded in the cohorts. Interestingly, it has been observed that, in general, women elicit higher antibody titers to infections and vaccinations [58]. However, lower antibody titers were also observed in non-human primates after HIV-1 protein vaccination [59], and it was observed that females had a lower frequency of breadth [15,38], which agrees with the observed findings in this study and could have been enhanced by the drug use.

Viral diversity within the first year of infection, another proposed marker for bNAb development, did not correlate with bNAb development. Interestingly, despite the lower overall prevalence of bNAb responses in the IDU group, more elite neutralizers were found in this group, with 6% of male IDUs qualifying as elite neutralizers compared to only 0.3% of MSM and 0% of female IDUs. When we analyzed the viral diversity of samples obtained in the first three months post-SC, we did find that all but one IDU demonstrated evidence consistent with a pattern associated with MVT. These findings are in concordance with other studies in which MVT has been observed in up to 60% of the IDU individuals [60,61]. Interestingly, the MSM elite neutralizer was the only MSM predicted to have MVT, suggesting that MVT might have contributed to the high level of bNAbs in this individual. The effect of very diverse sequences—for example, superinfection—on bNAb development is also still unclear, with some studies suggesting that superinfection might increase neutralization breadth [62,63], whereas others observed no such increase [54,64]. These conflicting results suggest that the role of viral diversity in the induction of bNAbs is probably a co-dependency factor.

## 5. Conclusions

In conclusion, we found that women infected with HIV-1 using contaminated needles developed bNAb responses less efficiently than men that attracted HIV-1 via the same transmission route. This difference is most likely multifactorial, where early viral diversity caused by multivariant transmission, CD4+ T cell count, viral load and drug use may all play a role. Therefore, the effect of gender on the development of bNAb responses is a factor that should be considered when designing vaccine efficacy trials.

## Figures and Tables

**Table 1:** Prevalence of broadly neutralizing antibody responses in HIV-1 infected MSM and IDUs.

	N	% Individuals with bNAb Responses (≥ 4 Viruses Neutralized with IC50 > 100)		% Elite Neutralizers (GMT > 500)	
MSM	299	27%	*p* = 0.1567	0.3%	*p* = 0.0354
IDU	85	19%		3.5%	
IDU-Male	50	20%	*p* = 0.7839	6.0%	*p* = 0.2647
IDU-Female	35	17%		0.0%	

**Table 2:** Factors associated with the development of broadly neutralizing antibodies in participants of the Amsterdam Cohort.

	Univariate Analysis		Multivariate Analysis*	
	*p*-Value	Effect	*p*-Value	Effect
Gender and route of transmission	0.002	−0.16	0.001	−0.12
CD4^+^ T cell count at setpoint	0.002	−0.16	0.017	−0.13
Viral load at setpoint	0.011	1.34	0.127	0.09

* The multivariable model was adjusted for the variables shown in the table.

**Table 3:** Factors associated with the presence of broadly neutralizing antibodies in participants of the Swiss 4.5k Screen.

		Univariable Analysis		Multivariable Analysis	
		*p*-Value	Effect	*p*-Value	Effect
Risk Group and Sex	MSM	1.5 × 10^−5^	−1.16	0.004	−0.77
	IDU-Male	reference		reference	
	IDU-Female	0.007	−1.06	0.006	−1.04
CD4^+^ T cell count		0.005	−0.31	0.100	−0.19
Viral load		0.019	0.33	4.5 × 10^−4^	0.51
Infection Time	Group 1	reference		reference	
	Group 3	2.3 × 10^−26^	3.40	2.8 × 10^−27^	3.44
	Group 5	3.1 × 10^−33^	3.72	2.1 × 10^−32^	3.76

Data reanalyzed from Rusert et al. [15]. The value “reference” shows that this group was used for comparison with the other groups. Group 1, low bNAb score; group 3, intermediate bNAb score; group 5, high bNAb score. The multivariable model was adjusted for the variables shown in the table.

## Supplementary Materials

The following are available online at https://www.mdpi.com/1999-4915/11/4/384/s1, Table S1: Serum neutralization capacity of MSM and IDU of the Amsterdam Cohort.
